# Software

**DOI:** 10.1155/S146392460000016X

**Published:** 2000


Journal of Automated Methods & Management in Chemistry, Vol. 22, No. 4 (July–August 2000) pp. 113

### Software review

### Automated dissolution testing interface software

Zymark announces the release of the Zymark/ChemStation interface software. When utilized in conjunction with software Version 2.1 for the MultiDose family of automated dissolution testing workstations and Version A.06.03 of Hewlett-Pakcard's ChemStation, which hosts a dissolution testing software add-on module, a direct link between the MultiDose or MultiDose Plus to the Hewlett-Packard ChemStation can be achieved. The ChemStation is configured with the Hewlett-Packard 8453 UV-visible dissolution testing system.

When on-line UV/VIS dissolution tests are run, these new software revisions allow for the total solution in fully unattended dissolution testing. All of the method information and analysis parameters generated by the MultiDose workstation are passed to the ChemStation, which also captures absorbance reading of standards and samples from the HP8453 UV/VIS spectrophotometer. These data, along with other parameters stored in a method file on the ChemStation, permit the calculation of dissolution rate release statistics. Final results are generated, printed and stored, while raw data are archived in a compliant fashion.

The Zymark MultiDose Plus is an advanced workstation-based system which fully automates dissolution tests employing:

- USP Apparatus I;
- USP Apparatus II;
- USP Apparatus II with capsule sinkers;
- Media Exchange, e.g. those performed on enteric-coated formulations, when run under USP Apparatus I.

The MultiDose Plus is an extension of Zymark's widely accepted MultiDose automated dissolution workstation, which fully automates USP Apparatus II dissolution tests. Both systems can fully automate up to eight batches without any manual intervention. The Hewlett-Packard ChemStation and HP8453 UV/VIS dissolution testing system, provide state-of-the-art spectroscopy and data reduction.

Zymark impacts the world by advancing the ability to discover, develop and market safe and effective drugs that can lengthen and improve the quality of life for everyone. Zymark's employees are active in more than 35 countries and their combined expertize in pharmaceutical analysis and laboratory automation makes Zymark the world's leading supplier in this application area.

*For more information contact: Sharon Correia, Director of Marketing Communications, Zymark Corporation, Zymark Center, Hopkinton, MA 01748, USA. Tel.: (508) 497-6403, e-mail: sharon.correia@zymark.com*

